# Effects of Exercise Training on the Autophagy-Related Muscular Proteins Expression in Ovariectomized Rats

**DOI:** 10.3389/fphys.2019.00735

**Published:** 2019-06-13

**Authors:** Weiquan Zhong, Xiangrong Shi, Honghua Yuan, Huimin Bu, Lianlian Wu, Renwei Wang

**Affiliations:** ^1^School of Kinesiology, Shanghai University of Sport, Shanghai, China; ^2^School of Medical Technology, Xuzhou Medical University, Xuzhou, China; ^3^Institute for Healthy Aging, University of North Texas Health Science Center, Fort Worth, TX, United States; ^4^Research Facility Center for Morphology, Xuzhou Medical University, Xuzhou, China; ^5^Department of Physiology, Xuzhou Medical University, Xuzhou, China; ^6^Laboratory Animal Center, Xuzhou Medical University, Xuzhou, China

**Keywords:** autophagy, body weight, ovariectomy, skeletal muscle, swimming exercise

## Abstract

Ovariectomy disrupts estrogen production and homeostasis. However, whether exercise training (ET) could counteract the ovariectomy-induced effect on muscular autophagy has remained elusive. This study examined muscular autophagy in ovariectomized (OVX) rats following 8 weeks of swimming ET. Here, 40 6-month-old female Sprague-Dawley rats were randomly divided into five groups: sham-operated control (Sham), OVX control (OVX), OVX with 60-min ET (OVX-60ET), 90-min ET (OVX-90ET), and 120-min ET (OVX-120ET) for 6 days/week. According to the results of Western blotting, the expression levels of autophagy-related proteins in the OVX gastrocnemius muscle, including mammalian target of rapamycin, uncoordinated 51-like kinase 1, Beclin-1, autophagy-related gene (Atg-7), and microtubule-associated protein light chains 3 were significantly decreased (all *P* < 0.05), while there was an elevation on the p62 level. ET appreciably mitigated the OVX-induced negative effects on muscle quality and the autophagy pathway, which seemed to be dependent on ET volume. The most optimal outcomes were observed in the OVX-90ET group. The OVX-120 group had an adversely augmented catabolic process associated with gastrocnemius muscle atrophy. In conclusion, the expression levels of autophagy proteins are decreased in OVX rats, which can be appreciably mitigated following 8 weeks of swimming ET.

## Introduction

Declination of ovarian function in aging women is accompanied by decreasing estrogen. Studies have reported that postmenopausal women are more susceptible to having obesity, cardiometabolic diseases, osteoporosis, and sarcopenia (Carr, 2003; Fu et al., 2009; Rector et al., 2011). Subsequently, hormone replacement therapy is taken as a beneficial intervention to provide balance against menopause. Estrogen supply has been utilized through modifications of autophagy in rodent studies to enhance insulin sensitivity (Kawakami et al., 2018) in order to maintain osteocyte viability (Florencio-Silva et al., 2018), promote recovery of neuromuscular function (Lin et al., 2016), protect skeletal muscle, and restore physical activity (Cabelka et al., 2019). However, menopause-induced morbidities are exacerbated by the high prevalence of physical inactivity associated with aging (Gomes et al., 2017), especially in older woman (Chan et al., 2018). However, the effects of exercise training (ET) on muscular autophagy in the absence of estrogen have remained elusive.

Skeletal muscles represent 40–45% of body mass in a healthy, lean individual (Janssen et al., 2000) and function to maintain the body’s posture and perform physical motion in addition to furnishing the body as the primary protein reservoir (Argiles et al., 2016). Declines in mass and strength of skeletal muscle resulting from sarcopenia are commonly associated with aging (Doherty, 2003) in postmenopausal women (Aubertin-Leheudre et al., 2008). This aging-induced problem has a series of resultant adverse health outcomes, including frailty, disability, and mortality (Walston, 2012). Previously, muscular autophagy has been noticed to contribute to regulate muscular function, atrophy, and hypertrophy (Schiaffino et al., 2013). Autophagy is an intracellular conserved catabolic process, in which long-lived proteins and damaged organelles are delivered and degraded in the lysosome (Parzych and Klionsky, 2014). Therefore, autophagy serves as a dynamic recycling system to keep the balance of the cellular renovation and homeostasis, which can be regulated by physical exercise (Ferraro et al., 2014; Tam and Siu, 2014). It has been reported that physical exercise stimulates autophagy in the skeletal muscles of mice, preventing the accumulation of damaged organelles and helping to maintain myofiber homeostasis (Grumati et al., 2011). Therefore, we hypothesized that endurance swimming ET activated muscular autophagy by promoting the expressions of muscular autophagy proteins in ovariectomized (OVX) rats.

In initiating autophagy process of skeletal muscles, the mechanistic (also known as “mammalian”) target of the rapamycin (mTOR) is presented as a key regulator that inhibits autophagy initiation (Tam and Siu, 2014) while it activates protein synthesis (Ferraro et al., 2014). During autophagy activation, mTOR dissociates from the uncoordinated 51-like kinase 1 (ULK1) complex and causes dephosphorylation and activation (Drummond et al., 2009; Goodman et al., 2011; Castets et al., 2013). Endurance physical exercise activates AMP-activated protein kinases (AMPK), which phosphorylates ULK1 and decreases mTOR-inhibited phosphorylation of ULK1 (Pagano et al., 2014). The activated ULK1 can initiate the autophagy process directly and indirectly through activating the phosphorylation of Beclin-1, further facilitating the initiation of muscular autophagy by detaching from Bcl-2 (Tam and Siu, 2014). The purpose of this study was to examine the effect of ET with different swimming durations or ET volume in training sessions on muscular autophagy in OVX rats. Therefore, the intracellular levels of mTOR, ULK1, and Beclin-1 of gastrocnemius muscle were analyzed. Moreover, proteins involved in the skeletal muscle autophagy signaling pathway, including autophagy-related gene (Atg-7), microtubule-associated protein light chains 3 (LC3I-II), and their targeted molecule p62, were compared since they have been shown to be modulated by ET (McConell et al., 2005; Ferraro et al., 2014; Saleem et al., 2014; Brandt et al., 2018). We postulated that ET would provide a beneficial influence on muscular autophagy (Broderick et al., 2005; Lira et al., 2013) and counteract or mitigate the OVX-induced negative effect on these proteins in gastrocnemius muscle.

It has been previously reported in a rodent model that wheel-running exercise for 4 weeks promoted protein expression of autophagy and increased autophagy flux in the plantaris muscle (Lira et al., 2013), and treadmill ET for 8 weeks upregulated an age-induced attenuation of gastrocnemius autophagy (Kim et al., 2013). Exhaustive running on a treadmill significantly increased autophagy marker LC3-II and decreased p62 in mouse soleus and deep quadriceps red muscles (Pagano et al., 2014). Swimming ET was selected in the present study because it has been proven to be effective with 60 min/training session, 5 sessions/week for 6 weeks (Gomes et al., 2006), or up to 90 min/training session, 5 sessions/week for 8 weeks (Iemitsu et al., 2002). Furthermore, it has been reported in a rat model that swimming training helps counteract the diabetes-induced muscle atrophy by suppressing autophagy process (Lee et al., 2012), mitigates dysfunctional autophagy, and provides antioxidant protection (Kou et al., 2017). Moreover, swimming ET provides an alternative to weight-bearing running exercise (Kawasaki et al., 2011) and has minimal risk of causing foot/leg injuries to the animals, which may be as beneficial as running ET (Nualnim et al., 2011). Three different ET session durations, i.e., 60, 90, and 120 min per session, 6 sessions/week for 8 weeks were selected in the present study to compare the influences of different ET volumes on muscular autophagy.

## Materials and Methods

### Animal Management

Here, 40 6-month-old female Sprague-Dawley rats (180–220 g) were provided by the Center of Experimental Animal, Xuzhou Medical University (Xuzhou, China). All rats were housed under a humidity (50 ± 10%) and temperature controlled (24 ± 1°C) room with 12:12 h light–dark cycle. All animals were allowed freely to access water and rodent chow throughout the study. The study protocol and experiment (Protocol #2016-036) were reviewed and approved by the Animal Ethics Committee of Xuzhou Medical University in accordance with the Guidelines for Ethical Conduct in the Care and Use of Animals.

After a week of acclimatization, 40 rats were subjected to a bilateral ovariectomy operation. Under anesthesia through intraperitoneal injection with 10% chloral hydrate (3.5 ml/kg), an incision of about 1.2 cm was made in each of the dorsal flank regions of the rat to expose the ovaries. The fallopian tubes were sutured at the most distal ends and the ovaries were excised for 32 rats. The other eight rats were subjected to the same surgical procedure without excising ovaries to serve as the Sham group (*n* = 8). The incisions were sutured and dressed with povidone iodine for 7 days (Reddy Nagareddy and Lakshmana, 2005). In addition, antibiotics (penicillin, 40 kU/kg wt) was administered via intramuscular injection for 3 consecutive days after the surgery. After another week of adaptive breeding following the surgical procedure without povidone iodine, the OVX rats were randomly divided into four groups (*n* = 8 for each): OVX group with no intervention (*n* = 8) and OVX rats which underwent ET for 60 min/day (OVX-60ET, *n* = 8), 90 min/day (OVX-90ET, *n* = 8), and 120 min/day (OVX-120ET, *n* = 8).

### Exercise Protocol

After 2 weeks of the operation and recovery from the surgery, animals in the ET group started to familiarize themselves with the swimming pool for 5–20 min daily for 5 consecutive days. Swimming ET was conducted in a cylindrical plastic barrel with diameter of 75 cm and height of 85 cm. Water was filled to a depth of 65 cm and water temperature was maintained at approximately 35°C. Additionally, four animals in a group were trained per barrel for about 5–20 min daily for 5 consecutive days to familiarize the setup and routine. Animals were dried with cotton towels after every swimming training session. After familiarization, animals in the OVX-60ET, OVX-90ET, and OVX-120ET groups began to perform 60-, 90-, and 120-min swimming exercise each day, respectively, for 6 days/week for 8 weeks. This protocol has been proved to be effective for promoting aerobic capacity of rats (Habibi et al., 2016). The Sham and OVX groups did not carry out any exercise. To minimize the acute exercise effect, exercise trained rats were euthanized ≥24 h after their last exercise session.

### Histological Assay

After obtaining the body weight, the animals were anesthetized with chloral hydrate. The completely anesthetized rats were decapitated to ensure death, and then the gastrocnemius muscles were isolated, harvested, weighed, and fixed with 4% paraformaldehyde solution. To investigate histological alterations, tissue samples from the gastrocnemius muscle were randomly selected and dehydrated through a graded series of alcohols and laid open before being embedded in paraffin. Serial sections were cut at 5 μm from three samples per group. Sections were then stained with hematoxylin–eosin (H&E) (Govindan et al., 2012). Each specimen was analyzed with an Olympus DP70 digital camera (Olympus, Tokyo, Japan) interfaced with a computer. The sections were observed under a light microscope at 40× magnification.

### Estrogen Measurement

Blood samples were collected and immediately chilled on ice in tubes containing 5 μl heparin. The samples were centrifuged at 4000 rpm for 15 min at 4°C and then stored at -80°C until analysis. Serum estrogen concentrations were determined using an E2 enzyme-linked immunosorbent kit (sensitivity range, 2–1000 pg/ml) (Nanjing Jiancheng Bioengineering Institute, Nanjing, China).

### Western Blot Analysis

The gastrocnemius skeletal muscle tissues were homogenized in a lysis buffer (Biyuntian, Haimen, China) with a polytron homogenizer and an ultrasonic processor on ice. The bicinchoninic acid (BCA) assay (Thermo Fisher Scientific, Waltham, MA, United States) was used according to the manufacturer’s instructions to quantify the level of protein in each sample to ensure equal protein loading. The protein samples were denatured with sodium dodecyl sulfate (SDS) loading buffer (5×) and separated by SDS–polyacrylamide gel electrophoresis (SDS–PAGE). Furthermore, 40 mg proteins along with a molecular weight protein marker (Thermo Fisher Scientific, Waltham, MA, United States) were subjected to SDS–PAGE using 7.5% acrylamide gel and electroblotted onto polyvinylidene fluoride (PVDF) membranes. The membrane was blocked with 5% non-fat milk in TBS containing 0.1% Tween 20 (TBS-T) and then probed at 4°C for 12 h with the primary antibodies against Beclin-1 (rabbit polyclonal, 1:1000; Cell Signaling Technology, Danvers, MA, United States), ULK1 (rabbit polyclonal, 1:300; Cell Signaling Technology, Danvers, MA, United States), Atg-7, mTOR, LC3, and p62/SQSTM1 (rabbit polyclonal, 1:1000; Proteintech Group Inc., Rosemont, IL, United States), and β-actin (mouse monoclonal, 1:5000; Proteintech Group Inc., Rosemont, IL, United States). After washing three times for 10 min with 0.1% TBS-T solution, the horseradish peroxidase (HRP)-conjugated secondary antibody (dilution of 1:4000 for goat anti-rabbit and dilution of 1:4000 for goat antimouse) was incubated at room temperature for 2 h, and signals were detected using Odyssey (Gene Company Ltd., Hong Kong, China). Band intensities were analyzed using Image J 1.25 software (National Institutes of Health, Bethesda, MD, United States) and the expression levels of all proteins were normalized to β-actin as described previously (Chen et al., 2016).

### Statistical Analysis

A paired *t*-test was applied to assess significant difference in body mass before and after the intervention. Two-way analysis of variance (ANOVA) was applied to test the significance of OVX factor and ET factor. *Post hoc* analysis with Tukey’s test was performed if the main factor had a significant influence. Statistical analyses were undertaken using SPSS 17.0 software (IBM, Armonk, NY, United States). All data were reported as mean ± standard error of the mean (SEM). Statistical significance was accepted at *P* ≤ 0.05.

## Results

### Body Mass

Baseline body mass (prior to ET) was not statistically different among the groups (Table 1). The Sham group had an increase in body mass (77 g) as a result of natural growth during the study; meanwhile, the OVX group had (141 g) an increase in body mass, in which more than 80% (*P* < 0.01) of that was in the Sham group, despite the fact that all animals accessed food and water freely without restriction. ET seemed to have a bifurcated effect on body mass. The OVX-60ET (*P* < 0.05) and OVX-90ET (*P* < 0.01) groups gained more weight compared with the Sham group, whereas the OVX-120ET group had no difference in the change of body weight compared with the Sham group. Furthermore, body weight of the OVX-120ET group was significantly lower compared with the OVX, OVX-60ET, and OVX-90ET groups.

**Table 1:** Body weight before and after 8 weeks of ET.

	Sham (*n* = 8)	OVX (*n* = 8)	OVX-60ET (*n* = 8)	OVX-90ET (*n* = 8)	OVX-120ET (*n* = 8)
Before (g)	198 ± 11	194 ± 10	204 ± 15	198 ± 11	197 ± 10
After (g)	275 ± 29	335 ± 21**	312 ± 31	329 ± 40*	269 ± 33^#^
Change (g)	77 ± 19	141 ± 27**	108 ± 20	131 ± 30**	72 ± 21^#^

*Sham, sham-operated control; OVX, ovariectomized control; OVX-60ET, OVX-90ET, and OVX-120ET denote ovariectomized rats with ET for 60, 90, and 120 min/day, respectively, 6 days/week for 8 weeks. ^∗^*P* < 0.05 vs. Sham, ^∗∗^*P* < 0.01 vs. Sham; ^#^*P* < 0.05 OVX-120ET vs. OVX, OVX-60RT, and OVX-90ET. Data are represented as the mean ± standard error of the mean (SEM) (*N* = 8 for all groups).*

### Estrogen Level and Uterus Weight

**FIGURE 1:**
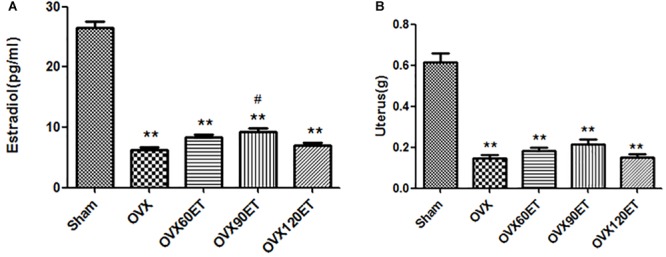
Effects of ovariectomy and swimming training on serum estradiol level (A) and uterus weight (B). estrogenOVX, ovariectomy; VX-60ET, ovariectomy with 60 min ET; OVX-90ET, ovariectomy with 90 min ET; OVX-120ET, ovariectomy with 120 min ET. Data are represented as mean ± standard error of the mean (SEM; *n* = 8 in each group). ^∗∗^*P <* 0.01 vs. Sham; ^#^*P* < 0.05 OVX-90ET vs. OVX.

Serum estradiol concentration was significantly (*P* < 0.01) lower after ovariectomy compared with the Sham group (Figure 1A). There was a significant difference (*P* < 0.05) in estradiol level between the OVX and OVX-90ET groups. Ovariectomy resulted in a significant reduction in uterus weight (Figure 1B). There was no remarkable difference in the OVX group with or without swimming ET.

### Muscle Mass

At the end of the experiment, the mass of gastrocnemius muscles was significantly greater in the OVX group than the Sham group. When the total body mass was normalized, the difference in muscle mass between the OVX group and the Sham group was not significant (Table 2). ET for 60 and 90 min showed a significant difference in the muscle mass compared with the Sham group. However, the muscle mass was not statistically different between the Sham and the OVX-120ET groups. There was no group difference after normalizing with the body mass.

**Table 2:** Gastrocnemius muscle mass and its ratio to body mass after 8 weeks of swimming ET.

	Sham (*n* = 8)	OVX (*n* = 8)	OVX-60ET (*n* = 8)	OVX-90ET (*n* = 8)	OVX-120ET (*n* = 8)	*P*-value	
						OVX factor	ET factor
MW (mg)	1565 ± 10	1929 ± 46**	1821 ± 50*	1895 ± 78**	1438 ± 67#	0.010	0.002
MW/BW (mg/g)	5.7 ± 0.1	5.8 ± 0.1	5.9 ± 0.2	5.8 ± 0.1	5.3 ± 0.1	0.702	0.447

*The ratio is calculated from muscle weight (MW) in milligrams/body weight (BW) in grams. OVX, ovariectomized rats; OVX-60ET, OVX-90ET, and OVX-120ET denote OVX rats with ET for 60, 90, and 120 min/day, respectively, 6 days/week for 8 weeks. *P*-value is the outcome of two-way ANOVA, i.e., ovariectomy (OVX) and ET factors. ^∗^ and ^∗∗^ denote *P* < 0.05 and *P* < 0.01, respectively, compared with Sham group and # indicates a significant difference at *P* < 0.01 compared with OVX, OVX-60ET, and OVX-90ET groups according to the *post hoc* analysis. Data are represented as the mean ± standard error of the mean (SEM) (*N* = 8 for all groups).*

### Histological Examination

**FIGURE 2:**
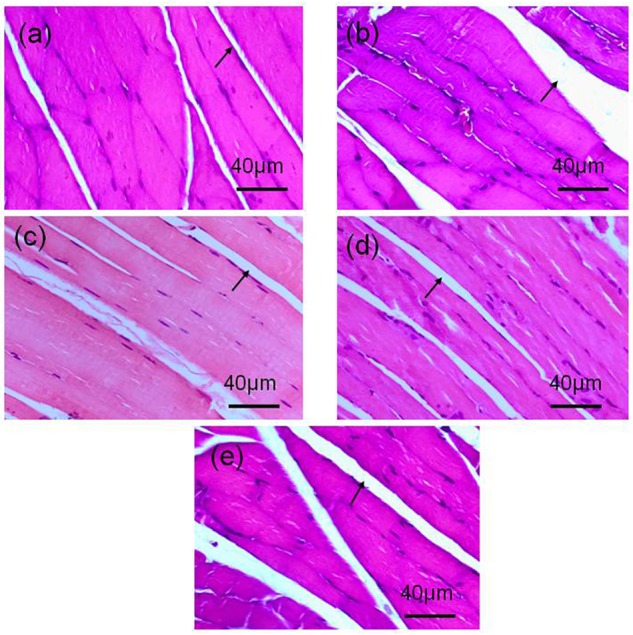
Histological examination on gastrocnemius muscle of rats. H&E staining images (magnification 400×, scale bar = 40 μm) from longitudinal view of gastrocnemius muscles of animals. **(a)** Sham-operated control, **(b)** ovariectomy control, **(c)** ovariectomy with ET 60 min, **(d)** 90 min, and **(e)** 120 min per session, 6 sessions/week for 8 weeks. There is appreciable fat accumulation in the gastrocnemius muscle of the animal following ovariectomy (indicated by arrow), which is effectively mitigated by ET.

There was increased intermuscular fat in the OVX group (Figure 2b) compared with the Sham group (Figure 2a). This fat infiltration and/or accumulation associated with OVX seemed to be appreciably mitigated by ET of 60 (Figure 2c), 90 (Figure 2d), or 120 min (Figure 2e). However, the diameter of individual muscle fibers in the OVX-120ET group was reduced. Compared with the OVX and OVX-120ET groups, the Sham, OVX-60ET, and OVX-90ET groups showed adequately preserved myofibers with clear striations and peripheral myonuclei. Moreover, the nuclear density was appreciably increased in the OVX-90ET group (Figure 2d).

### Autophagy Proteins

**FIGURE 3:**
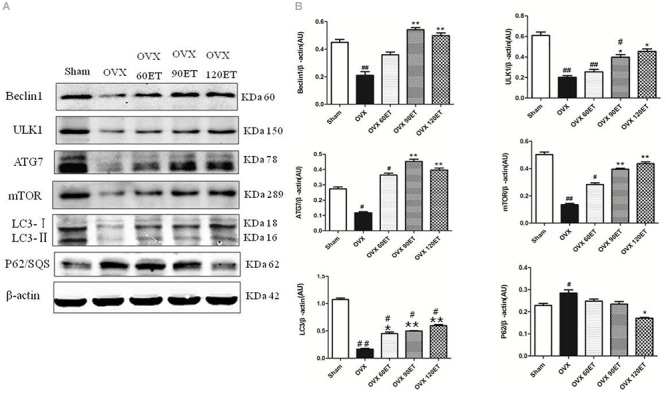
Immunoblots and group data of autophagy proteins. **(A)** From top to bottom shows representative immunoblots on the expressions of Beclin-1, ULK1, Atg-7, LC3-I/II, β-actin, mTOR, P62/sequestosome, and β-actin, respectively, for animals undergoing sham surgery, ovariectomy (OVX), ovariectomy with ET for 60 min (OVX-60ET), 90 min (OVX-90ET), and 120 min (OVX-120ET) per session, 6 sessions/week for 8 weeks. Panel **(B)** summarizes data as the mean ± standard error of the mean (SEM). Whole muscle protein analysis is corrected for loading with β-actin. ^#^*P* < 0.05 and ^##^*P* < 0.01 vs. Sham group. ^∗^*P* < 0.05 and ^∗∗^*P* < 0.01 vs. OVX group.

Figure 3 illustrates the results of Western blotting (Figure 3A) and group data (Figure 3B) of the expression levels of Beclin-1, ULK1, Atg-7, LC3-I/II, mTOR, and p62. Ovariectomy significantly depressed the expression levels of all autophagy proteins Beclin-1, ULK1, Atg-7, LC3-I/II, and mTOR, except for p62 protein which was significantly elevated. ET tended to offset the OVX effect on these autophagy proteins in a dose–response or ET-dependent manner, minimizing the differences in the expression levels of these proteins between the ET group and the Sham group. However, the intracellular levels of ULK and LC3 in gastrocnemius muscle remained significantly lower in the OVX group compared with the Sham group.

## Discussion

The present study examined the effects of 8-week swimming ET on key proteins of muscular autophagy and muscle morphological changes following ovariectomy in rats. Ovariectomy significantly reduced serum estrogen concentration and uterus weight. ET effectively counteracted the ovariectomy-induced negative influences on the expression levels of mTOR, ULK1, Beclin-1, Atg-7, LC3, and p62. Although there was a dose–response trend in exercise time per training session on muscular autophagy, in terms of the muscle mass and quality, and autophagy proteins, the OVX animals with 90 min exercise per training session seemed to be most optimal compared with the OVX-60ET and OVX-120ET groups.

### Body Mass and Muscle Mass

It has been reported that there is an increase in accumulation of adipose tissue mass without any change in energy intake in OVX animals (Rogers et al., 2009). The mechanisms for this body fat accumulation are likely associated with ovariectomy-induced decrease in energy expenditure (Rogers et al., 2009), upregulation of adipogenic genes (Rogers et al., 2009; Frechette et al., 2015), and an increase in feeding efficiency with impaired glucose homeostasis (Dornellas et al., 2018). The present study confirmed that the increased body mass was 80% greater in the OVX group compared with the Sham group (Table 1). Furthermore, the gastrocnemius muscle mass was also significantly greater (+23%) in the OVX group than the Sham group (Table 2), which was likely attributable to fat infiltration and/or accumulation within the skeletal muscle (Figure 2). Greater fat infiltration/accumulation in skeletal muscles commonly occurs with the gain of excessive weight or obesity (Kelley et al., 1991). Subsequently, the quality of skeletal muscles in OVX animals declines, correlating with muscle weakness (Goodpaster et al., 2000) and impaired mobility (Visser et al., 2002).

Additionally, 8-week swimming training in all three training regimens with different exercise durations seemed to prevent this ovariectomy-induced fat accumulation in the muscle. Furthermore, the gastrocnemius muscle mass was significantly increased in the OVX group following ET for 60 and 90 min/day, 6 days/week for 8 weeks (i.e., OVX-60ET and OVX-90ET groups). Nonetheless, the gastrocnemius muscle mass of the OVX120ET group was significantly lower compared with the OVX-60ET and OVX-90ET groups (Table 2). These data suggested that endurance ET could appreciably augment muscle mass of the OVX animals if the training volume was moderate and adequate. High training volume with prolonged exercise duration, i.e., 120 min/training session in the OVX-120ET group, had an adverse effect on the skeletal muscle mass as was evidenced by the muscle atrophy associated with the diminished size of individual muscle fibers (Figure 2).

### Autophagy Following Ovariectomy

Autophagy is essential for maintaining muscle mass and quality, as well as preventing muscle atrophy and dysfunction (Masiero et al., 2009). Compared with the Sham group, OVX animals showed significant decreases in the expression levels of all key autophagy proteins, including Beclin-1, ULK1, ATG-7, LC3, and mTOR, except for P62 (Figure 3). Functioning as a key regulator in muscular autophagy, mTOR phosphorylates and inhibits the ULK1/2 complex. Thus, mTOR exerts an inhibitory influence on the initiation of the autophagy pathway and promotes anabolic process or cell growth with adequate supply of nutrients (Dunlop and Tee, 2014). Ovarian hormones have been known to activate mTOR via the Akt signaling pathway (Pedram et al., 2002; Patten et al., 2004) and diminish mTOR-mediated protein anabolic metabolism (Dunlop and Tee, 2014). This could be a mechanism for the reduced muscle mass along with the increased fat accumulation following ovariectomy without ET observed in the OVX group. The depressed expression of mTOR can minimize or remove the inhibitory influence on autophagy, which is concomitantly suppressed by ovariectomy as well.

Autophagy initiation is controlled through activation of the ULK1 complex and is associated with the inhibitory influence of mTOR. Although there was a significant decrease in the intracellular level of mTOR observed in the OVX group, the expression levels of ULK and the downstream proteins in muscular autophagy cascade, including Beclin-1, LC3 and ATG-7, were all significantly decreased in the OVX group (Figure 3). These decreased expressions of autophagy proteins in the present study indicated that a basal level of autophagy–lysosomal process with protein recycle and homeostasis in the gastrocnemius muscle could be suppressed, and catabolic process via the autophagy–lysosome pathway was compromised following ovariectomy as well.

Sequestosome 1 (SQSTEM1) or p62 is an important cargo receptor involved in the autophagy lysosomal pathway and ubiquitin-proteasome system (Liu et al., 2016). The intracellular level of p62 can be modified by two factors (i) the transcription, which is upregulated by oxidative stress, proteasome inhibition, and starvation (Myeku and Figueiredo-Pereira, 2011; Sahani et al., 2014), and (ii) degradation, inversely correlating with autophagy process (Komatsu and Ichimura, 2010; Rogov et al., 2014). Thus, suppression of autophagy is always accompanied by a marked accumulation of p62. In the present study, the expression level of p62 was significantly higher in the OVX group compared with the Sham group, while the expression levels of Beclin-1, ULK1, ATG-7, LC3, and mTOR were all significantly lower than ovariectomy. Taken together, these data suggested that lack of estrogen hormones following ovariectomy could suppress the muscular autophagy–lysosomal process.

### Autophagy Following Exercise Training

Here, 8-week swimming ET effectively counteracted the OVX-induced suppressions of autophagy proteins. The intracellular levels of Beclin-1, Atg-7, and p62 of the gastrocnemius muscle in all training groups, i.e., OVX-60ET, OVX-90ET, and OVX-120ET, were not notably different compared with these proteins in the Sham group. Moreover, there was no significant difference in the expression level of mTOR of the gastrocnemius muscle among the OVX-90ET, OVX-120ET, and Sham groups. These data suggested that ET could be applied to mitigate the inhibitory influence (due to lack of estrogen hormones) on the autophagy pathway. In addition, this effect seemed to be training volume-dependent.

Making a comparison among three training regimens with different exercise durations, i.e., OVX-60ET, OVX-90ET, and OVX-120ET, it was revealed that the 90-min training session was the most optimal. In the present study, the expression levels of mTOR, ULK1, and LC3 were lower in the OVX-60ET group compared with the Sham group, although they showed a trend of recovery. However, the expression levels of these proteins were not statistically different between the OVX-90ET and Sham groups. Although the OVX120ET group had similar intracellular levels of all these proteins compared with the OVX-90ET or Sham group, the p62 level of the OVX-120ET was appreciably lower than that in the OVX group. This observation indicated that the high training volume of the OVX120ET group could augment the autophagy–lysosomal pathway and catabolic process in the animals following ovariectomy, which consequently led to the atrophy observed in the gastrocnemius muscle (Figure 2 and Table 2). A previous study suggested that 1 h after an acute bout of high-intensity exercise, rather than low-intensity exercise, the p62 level was significantly suppressed compared to the Sham group, indicating an increased autophagy–lysosomal signaling pathway (Schwalm et al., 2015). In the present study, ≥24 h from the last ET, the expression of p62 remained lower in the OVX120 group. Collectively, these data suggested that exercise or training at higher intensity or longer duration could adversely activate autophagy flex and negatively influence protein anabolic process.

### Study Limitations and Perspectives

A major limitation of the study is that there was no quantitative histological assay for the gastrocnemius muscles in the animals. Differences in lean body mass and/or fat mass with and without estrogens between swimming and running ET should be further studied. Autophagy is critical in maintaining muscle mass and quality. Deficiency of estrogen in animals following ovariectomy shows a suppressed muscular autophagy pathway, which may lead to a disruption of intracellular homeostasis involved in the activations of the catabolic process and anabolic process. This may be a mechanism for the OVX animals, resulting in the increased fat infiltration/accumulation in the skeletal muscle. This mechanism explains why aging women have declined muscle quality or strength and increased frailty or falling incidence. Swimming ET with moderate exercise volume helps counteract ovariectomy-induced suppression on the muscular autophagy process and mitigates fat accumulation in the muscle. However, high training volume can adversely augment muscular autophagy and cause muscle atrophy.

## Conclusion

In conclusion, the expression levels of autophagy proteins are decreased in OVX rats, which can be appreciably mitigated following 8 weeks of swimming ET.

## Data Availability

The raw data supporting the conclusions of this manuscript will be made available by the authors, without undue reservation, to any qualified researcher.

## Ethics Statement

The study protocols and experiments were reviewed and approved by the Animal Ethics Committee of Xuzhou Medical University, which conformed to the Guidelines for Ethical Conduct in the Care and Use of Animals (Protocol #2016-036).

## Author Contributions

WZ contributed to the hypothesis development, data collection, analysis and interpretation, and manuscript preparation. XS contributed to data interpretation and manuscript preparation. HY, HB, and LW contributed to the training of the rats and the data collection. RW contributed to the hypothesis development, data analysis and interpretation, and manuscript preparation.

## Conflict of Interest Statement

The authors declare that the research was conducted in the absence of any commercial or financial relationships that could be construed as a potential conflict of interest.

## References

[B1] Argiles J. M. Campos N. Lopez-Pedrosa J. M. Rueda R. Rodriguez-Manas L. (2016). Skeletal muscle regulates metabolism via interorgan crosstalk: roles in health and disease. *J. Am. Med. Dir. Assoc.* 17 789–796. 10.1016/j.jamda.2016.04.019 27324808

[B2] Aubertin-Leheudre M. Lord C. Labonte M. Khalil A. Dionne I. J. (2008). Relationship between sarcopenia and fracture risks in obese postmenopausal women. *J. Women Aging* 20 297–308. 10.1080/08952840801984964 18983113

[B3] Brandt N. Gunnarsson T. P. Bangsbo J. Pilegaard H. (2018). Exercise and exercise training-induced increase in autophagy markers in human skeletal muscle. *Physiol. Rep.* 6:e13651. 10.14814/phy2.13651 29626392 PMC5889490

[B4] Broderick T. L. Poirier P. Gillis M. (2005). Exercise training restores abnormal myocardial glucose utilization and cardiac function in diabetes. *Diabetes Metab. Res. Rev.* 21 44–50. 10.1002/dmrr.479 15386820

[B5] Cabelka C. A. Baumann C. W. Collins B. C. Nash N. Le G. Lindsay A. (2019). Effects of ovarian hormones and estrogen receptor alpha on physical activity and skeletal muscle fatigue in female mice. *Exp. Gerontol.* 115 155–164. 10.1016/j.exger.2018.11.003 30415069 PMC6331238

[B6] Carr M. C. (2003). The emergence of the metabolic syndrome with menopause. *J. Clin. Endocrinol. Metab.* 88 2404–2411. 10.1210/jc.2003-030242 12788835

[B7] Castets P. Lin S. Rion N. Di Fulvio S. Romanino K. Guridi M. (2013). Sustained activation of mTORC1 in skeletal muscle inhibits constitutive and starvation-induced autophagy and causes a severe, late-onset myopathy. *Cell Metab.* 17 731–744. 10.1016/j.cmet.2013.03.015 23602450

[B8] Chan Y. Y. Sooryanarayana R. Mohamad Kasim N. Lim K. K. Cheong S. M. Kee C. C. (2018). Prevalence and correlates of physical inactivity among older adults in Malaysia: Findings from the National Health and Morbidity Survey (NHMS) 2015. *Arch. Gerontol. Geriatr.* 81 74–83. 10.1016/j.archger.2018.11.012 30521992

[B9] Chen R. J. Wang Z. Z. Yuan H. H. Zhang T. Y. Fu Z. X. Hu A. K. (2016). Hypodermin A improves survival of skin allografts. *J. Surg. Res.* 203 15–21. 10.1016/j.jss.2016.03.037 27338529

[B10] Doherty T. J. (2003). Invited review: aging and sarcopenia. *J. Appl. Physiol.* 95 1717–1727. 10.1152/japplphysiol.00347.2003 12970377

[B11] Dornellas A. P. S. Boldarine V. T. Pedroso A. P. Carvalho L. O. T. de Andrade I. S. Vulcani-Freitas T. M. (2018). High-fat feeding improves anxiety-type behavior induced by ovariectomy in rats. *Front. Neurosci.* 12:557. 10.3389/fnins.2018.00557 30233288 PMC6129615

[B12] Drummond M. J. Fry C. S. Glynn E. L. Dreyer H. C. Dhanani S. Timmerman K. L. (2009). Rapamycin administration in humans blocks the contraction-induced increase in skeletal muscle protein synthesis. *J. Physiol.* 587(Pt 7) 1535–1546. 10.1113/jphysiol.2008.163816 19188252 PMC2678224

[B13] Dunlop E. A. Tee A. R. (2014). mTOR and autophagy: a dynamic relationship governed by nutrients and energy. *Semin. Cell Dev. Biol.* 36 121–129. 10.1016/j.semcdb.2014.08.006 25158238

[B14] Ferraro E. Giammarioli A. M. Chiandotto S. Spoletini I. Rosano G. (2014). Exercise-induced skeletal muscle remodeling and metabolic adaptation: redox signaling and role of autophagy. *Antioxid. Redox Signal.* 21 154–176. 10.1089/ars.2013.5773 24450966 PMC4048572

[B15] Florencio-Silva R. Sasso G. R. S. Sasso-Cerri E. Simoes M. J. Cerri P. S. (2018). Effects of estrogen status in osteocyte autophagy and its relation to osteocyte viability in alveolar process of ovariectomized rats. *Biomed. Pharmacother.* 98 406–415. 10.1016/j.biopha.2017.12.089 29276969

[B16] Frechette D. M. Krishnamoorthy D. Adler B. J. Chan M. E. Rubin C. T. (2015). Diminished satellite cells and elevated adipogenic gene expression in muscle as caused by ovariectomy are averted by low-magnitude mechanical signals. *J. Appl. Physiol.* 119 27–36. 10.1152/japplphysiol.01020.2014 25930028 PMC4491530

[B17] Fu M. H. Maher A. C. Hamadeh M. J. Ye C. Tarnopolsky M. A. (2009). Exercise, sex, menstrual cycle phase, and 17beta-estradiol influence metabolism-related genes in human skeletal muscle. *Physiol. Genomics* 40 34–47. 10.1152/physiolgenomics.00115.2009 19808840

[B18] Gomes M. Figueiredo D. Teixeira L. Poveda V. Paul C. Santos-Silva A. (2017). ”Physical inactivity among older adults across Europe based on the SHARE database. *Age Ageing* 46 71–77. 10.1093/ageing/afw165 28181637 PMC6402309

[B19] Gomes R. J. de Mello MA Caetano F. H. Sibuya C. Y. Anaruma C. A. Rogatto G. P. (2006). Effects of swimming training on bone mass and the GH/IGF-1 axis in diabetic rats. *Growth Horm. IGF Res.* 16 326–331. 10.1016/j.ghir.2006.07.003 17011807

[B20] Goodman C. A. Mayhew D. L. Hornberger T. A. (2011). Recent progress toward understanding the molecular mechanisms that regulate skeletal muscle mass. *Cell Signal.* 23 1896–1906. 10.1016/j.cellsig.2011.07.013 21821120 PMC3744211

[B21] Goodpaster B. H. Kelley D. E. Thaete F. L. He J. Ross R. (2000). Skeletal muscle attenuation determined by computed tomography is associated with skeletal muscle lipid content. *J. Appl. Physiol.* 89 104–110. 10.1152/jappl.2000.89.1.104 10904041

[B22] Govindan S. McElligott A. Muthusamy S. Nair N. Barefield D. Martin J. L. (2012). Cardiac myosin binding protein-C is a potential diagnostic biomarker for myocardial infarction. *J. Mol. Cell Cardiol.* 52 154–164. 10.1016/j.yjmcc.2011.09.011 21971072 PMC3246118

[B23] Grumati P. Coletto L. Schiavinato A. Castagnaro S. Bertaggia E. Sandri M. (2011). Physical exercise stimulates autophagy in normal skeletal muscles but is detrimental for collagen VI-deficient muscles. *Autophagy* 7 1415–1423. 10.4161/auto.7.12.17877 22024752 PMC3288016

[B24] Habibi P. Alihemmati A. NourAzar A. Yousefi H. Mortazavi S. Ahmadiasl N. (2016). Expression of the Mir-133 and Bcl-2 could be affected by swimming training in the heart of ovariectomized rats. *Iran J. Basic Med. Sci.* 19 381–387. 27279981 PMC4887710

[B25] Iemitsu M. Miyauchi T. Maeda S. Tanabe T. Takanashi M. Irukayama-Tomobe Y. (2002). Aging-induced decrease in the PPAR-alpha level in hearts is improved by exercise training. *Am. J. Physiol. Heart Circ. Physiol.* 283 H1750–H1760. 12384451 10.1152/ajpheart.01051.2001

[B26] Janssen I. Heymsfield S. B. Baumgartner R. N. Ross R. (2000). Estimation of skeletal muscle mass by bioelectrical impedance analysis. *J. Appl. Physiol.* 89 465–471. 10.1152/jappl.2000.89.2.465 10926627

[B27] Kawakami M. Yokota-Nakagi N. Uji M. Yoshida K. I. Tazumi S. P. D. Takamata A. (2018). Estrogen replacement enhances insulin-induced AS160 activation and improves insulin sensitivity in ovariectomized rats. *Am. J. Physiol. Endocrinol. Metab.* 315 E1296–E1304. 10.1152/ajpendo.00131.2018 30179516

[B28] Kawasaki T. Sullivan C. V. Ozoe N. Higaki H. Kawasaki J. (2011). A long-term, comprehensive exercise program that incorporates a variety of physical activities improved the blood pressure, lipid and glucose metabolism, arterial stiffness, and balance of middle-aged and elderly Japanese. *Hypertens. Res.* 34 1059–1066. 10.1038/hr.2011.81 21753777

[B29] Kelley D. E. Slasky B. S. Janosky J. (1991). Skeletal muscle density: effects of obesity and non-insulin-dependent diabetes mellitus. *Am. J. Clin. Nutr.* 54 509–515. 10.1093/ajcn/54.3.509 1877507

[B30] Kim Y. A. Kim Y. S. Oh S. L. Kim H. J. Song W. (2013). Autophagic response to exercise training in skeletal muscle with age. *J. Physiol. Biochem.* 69 697–705. 10.1007/s13105-013-0246-7 23471597

[B31] Komatsu M. Ichimura Y. (2010). Physiological significance of selective degradation of p62 by autophagy. *FEBS Lett.* 584 1374–1378. 10.1016/j.febslet.2010.02.017 20153326

[B32] Kou X. Li J. Liu X. Chang J. Zhao Q. Jia S. (2017). Swimming attenuates d-galactose-induced brain aging via suppressing miR-34a-mediated autophagy impairment and abnormal mitochondrial dynamics. *J. Appl. Physiol.* 122 1462–1469. 10.1152/japplphysiol.00018.2017 28302704

[B33] Lee Y. Kim J. H. Hong Y. Lee S. R. Chang K. T. Hong Y. (2012). Prophylactic effects of swimming exercise on autophagy-induced muscle atrophy in diabetic rats. *Lab. Anim. Res.* 28 171–179. 10.5625/lar.2012.28.3.171 23091517 PMC3469845

[B34] Lin C. W. Chen B. Huang K. L. Dai Y. S. Teng H. L. (2016). Inhibition of autophagy by estradiol promotes locomotor recovery after spinal cord injury in rats. *Neurosci. Bull.* 32 137–144. 10.1007/s12264-016-0017-x 26924807 PMC5563739

[B35] Lira V. A. Okutsu M. Zhang M. Greene N. P. Laker R. C. Breen D. S. (2013). Autophagy is required for exercise training-induced skeletal muscle adaptation and improvement of physical performance. *FASEB J.* 27 4184–4193. 10.1096/fj.13-228486 23825228 PMC4046188

[B36] Liu W. J. Ye L. Huang W. F. Guo L. J. Xu Z. G. Wu H. L. (2016). p62 links the autophagy pathway and the ubiqutin-proteasome system upon ubiquitinated protein degradation. *Cell Mol. Biol. Lett.* 21:29. 10.1186/s11658-016-0031-z 28536631 PMC5415757

[B37] Masiero E. Agatea L. Mammucari C. Blaauw B. Loro E. Komatsu M. (2009). Autophagy is required to maintain muscle mass. *Cell Metab.* 10 507–515. 10.1016/j.cmet.2009.10.008 19945408

[B38] McConell G. K. Lee-Young R. S. Chen Z. P. Stepto N. K. Huynh N. N. Stephens T. J. (2005). Short-term exercise training in humans reduces AMPK signalling during prolonged exercise independent of muscle glycogen. *J. Physiol.* 568(Pt 2) 665–676. 10.1113/jphysiol.2005.089839 16051629 PMC1474728

[B39] Myeku N. Figueiredo-Pereira M. E. (2011). Dynamics of the degradation of ubiquitinated proteins by proteasomes and autophagy: association with sequestosome 1/p62. *J. Biol. Chem.* 286 22426–22440. 10.1074/jbc.M110.149252 21536669 PMC3121389

[B40] Nualnim N. Barnes J. N. Tarumi T. Renzi C. P. Tanaka H. (2011). Comparison of central artery elasticity in swimmers, runners, and the sedentary. *Am. J. Cardiol.* 107 783–787. 10.1016/j.amjcard.2010.10.062 21247521

[B41] Pagano A. F. Py G. Bernardi H. Candau R. B. Sanchez A. M. (2014). Autophagy and protein turnover signaling in slow-twitch muscle during exercise. *Med. Sci. Sports Exerc.* 46 1314–1325. 10.1249/MSS.0000000000000237 24389528

[B42] Parzych K. R. Klionsky D. J. (2014). An overview of autophagy: morphology, mechanism, and regulation. *Antioxid. Redox Signal.* 20 460–473. 10.1089/ars.2013.5371 23725295 PMC3894687

[B43] Patten R. D. Pourati I. Aronovitz M. J. Baur J. Celestin F. Chen X. (2004). 17beta-estradiol reduces cardiomyocyte apoptosis in vivo and in vitro via activation of phospho-inositide-3 kinase/Akt signaling. *Circ. Res.* 95 692–699. 10.1161/01.res.0000144126.57786.89 15345655

[B44] Pedram A. Razandi M. Aitkenhead M. Hughes C. C. Levin E. R. (2002). Integration of the non-genomic and genomic actions of estrogen. membrane-initiated signaling by steroid to transcription and cell biology. *J. Biol. Chem.* 277 50768–50775. 10.1074/jbc.m210106200 12372818

[B45] Rector R. S. Uptergrove G. M. Morris E. M. Borengasser S. J. Laughlin M. H. Booth F. W. (2011). Daily exercise vs. caloric restriction for prevention of nonalcoholic fatty liver disease in the OLETF rat model. *Am. J. Physiol. Gastrointest Liver Physiol.* 300 G874–G883. 10.1152/ajpgi.00510.2010 21350190 PMC3094141

[B46] Reddy Nagareddy P. Lakshmana M. (2005). Assessment of experimental osteoporosis using CT-scanning, quantitative X-ray analysis and impact test in calcium deficient ovariectomized rats. *J. Pharmacol. Toxicol. Methods* 52 350–355. 10.1016/j.vascn.2005.06.001 15996488

[B47] Rogers N. H. Perfield J. W. II Strissel K. J. Obin M. S. Greenberg A. S. (2009). Reduced energy expenditure and increased inflammation are early events in the development of ovariectomy-induced obesity. *Endocrinology* 150 2161–2168. 10.1210/en.2008-1405 19179442 PMC2671894

[B48] Rogov V. Dotsch V. Johansen T. Kirkin V. (2014). Interactions between autophagy receptors and ubiquitin-like proteins form the molecular basis for selective autophagy. *Mol. Cell.* 53 167–178. 10.1016/j.molcel.2013.12.014 24462201

[B49] Sahani M. H. Itakura E. Mizushima N. (2014). Expression of the autophagy substrate SQSTM1/p62 is restored during prolonged starvation depending on transcriptional upregulation and autophagy-derived amino acids. *Autophagy* 10 431–441. 10.4161/auto.27344 24394643 PMC4077882

[B50] Saleem A. Carter H. N. Hood D. A. (2014). p53 is necessary for the adaptive changes in cellular milieu subsequent to an acute bout of endurance exercise. *Am. J. Physiol. Cell Physiol.* 306 C241–C249. 10.1152/ajpcell.00270.2013 24284795 PMC3919998

[B51] Schiaffino S. Dyar K. A. Ciciliot S. Blaauw B. Sandri M. (2013). Mechanisms regulating skeletal muscle growth and atrophy. *FEBS J.* 280 4294–4314. 10.1111/febs.12253 23517348

[B52] Schwalm C. Jamart C. Benoit N. Naslain D. Premont C. Prevet J. (2015). Activation of autophagy in human skeletal muscle is dependent on exercise intensity and AMPK activation. *FASEB J.* 29 3515–3526. 10.1096/fj.14-267187 25957282

[B53] Tam B. T. Siu P. M. (2014). Autophagic cellular responses to physical exercise in skeletal muscle. *Sports Med.* 44 625–640. 10.1007/s40279-013-0140-z 24549475

[B54] Visser M. Kritchevsky S. B. Goodpaster B. H. Newman A. B. Nevitt M. Stamm E. (2002). Leg muscle mass and composition in relation to lower extremity performance in men and women aged 70 to 79: the health, aging and body composition study. *J. Am. Geriatr. Soc.* 50 897–904. 10.1046/j.1532-5415.2002.50217.x 12028178

[B55] Walston J. D. (2012). Sarcopenia in older adults. *Curr. Opin. Rheumatol.* 24 623–627. 22955023 10.1097/BOR.0b013e328358d59b PMC4066461

